# SS31 Ameliorates Podocyte Injury via Inhibiting OMA1-Mediated Hydrolysis of OPA1 in Diabetic Kidney Disease

**DOI:** 10.3389/fphar.2021.707006

**Published:** 2022-01-31

**Authors:** Qianqian Yang, Wenjia Xie, Xiao Wang, Jing Luo, Yang Zhou, Hongdi Cao, Qi Sun, Lei Jiang, Junwei Yang

**Affiliations:** Center for Kidney Disease, 2nd Affiliated Hospital, Nanjing Medical University, Nanjing, China

**Keywords:** SS31, mitochondria, Oma1, OPA1, podocyte, diabetic nephropathy

## Abstract

Diabetic kidney disease (DKD) is currently one of the leading causes of end-stage renal disease (ESRD). Mitochondrial dysfunction in podocyte is involve in DKD development. However, whether early mitochondrial stabilization delays or reverses DKD progression has not been elucidated. SS31 is a novel tetrapeptide compound that targets the inner mitochondrial membrane and protects mitochondria by reducing ROS and inhibiting cardiolipin oxidation. Our study discovered that SS31 might have a long-term podocyte protection in DKD. In this study, we examined the glomerular pathological damage and proteinuria at different stages of diabetes. Results revealed that podocyte mitochondrial injury appeared at the early stage of DKD. Early treatment with SS31 could protect podocyte and alleviate the development of DKD via inhibiting OMA1-mediated hydrolysis of OPA1. Those data indicate that SS31 might be a promising agent in delaying the development of DKD and OMA1-mediated hydrolysis of OPA1 in mitochondria, and SS31 is a novel therapeutic target for the treatment of DKD.

## Introduction

Diabetic kidney disease (DKD) is currently one of the leading causes of end-stage renal disease (ESRD) and is the strongest single predictor of mortality in diabetic patients (Reidy et al., 2014). Accumulated evidences suggest that podocyte damage plays a vital role in DKD progression (Bose et al., 2017). Kidney biopsy of Type 1 diabetes mellitus (T1DM) and T2DM shows that podocyte number is highly correlated with proteinuria and acts as an important factor in predicting disease progression (White et al., 2002). As a type of terminally differentiated epithelial cells, podocytes have a limited potential in self-repair and regeneration and are sensitive to various injuries. Thus, effective protection of podocytes is an important strategy for the treatment of DKD.

Podocytes rely on glycolysis and mitochondrial oxidative phosphorylation for ATP synthesis, among which mitochondrial respiration accounts for 77% (Abe et al., 2010). It has been reported that mitochondrial abnormalities are involved in a variety of podocyte injury models (Guan et al., 2015; Szeto et al., 2016; Qi et al., 2017; Fujii et al., 2020), meanwhile mitochondria-targeted drugs significantly alleviate podocyte injury, indicating that mitochondrial homeostasis plays an important role in podocytes (Szeto, 2017). Mitochondrial homeostasis involves mitochondrial biogenesis, mitochondrial dynamics, mitochondrial distribution, mitophagy and mitochondrial DNA content. Among these, mitochondrial dynamics, the balance of mitochondrial fusion and fission, has a vital role in maintaining mitochondrial homeostasis (Cerveny et al., 2007; Chan, 2012; Mishra and Chan, 2016; Wai and Langer, 2016). Enough evidences show that abnormal mitochondrial dynamics is widely involved in podocyte injury (Wang et al., 2012; Yuan et al., 2018; Ma et al., 2019; Chen et al., 2020), and might be an important target for the treatment of podocyte diseases (Ayanga et al., 2016; Qin et al., 2019). The dynamin-like GTPase OPA1 is a crucial fusion protein in mitochondrial inner membrane. In addition, OPA1 also participates in mitochondrial cristae morphogenesis, apoptosis, and mitochondrial respiration (Olichon et al., 2006). OMA1, a mitochondrial inner membrane zinc metalloprotease, is involved in the proteolysis of OPA1 during stress and apoptosis (Ehses et al., 2009). Under physiological conditions, OMA1 is dormant, but rapidly activated upon mitochondrial dysfunctions (MacVicar and Langer, 2016). Thus, OPA1 and OMA1 play an important role in stabilizing mitochondria. SS31 is a cell-permeable tetrapeptide that selectively targets the inner mitochondrial membrane. Previous studies identified SS31 as a mitochondria-targeted antioxidant (Zhao et al., 2004). Recent research reveals that SS31 selectively interacts with cardiolipin and inhibits cardiolipin peroxidation, thus promoting ATP synthesis and reducing proton leak as well as ROS production (Szeto et al., 2011; Birk et al., 2013). Due to the suppression of cardiolipin peroxidation,SS-31 has shown remarkable efficacy in diverse animal disease models associated with bioenergetic failure, including ischaemia-reperfusion injury, heart failure, skeletal muscle atrophy and neurodegenerative diseases(Yang et al., 2009; Min et al., 2011; Szeto and Schiller, 2011; Kloner et al., 2012; Sloan et al., 2012; Dai et al., 2013; Talbert et al., 2013). The protective role of SS31 was also found in kidney in the progressing of acute kidney injury (AKI), DKD or aging. Mechanically, SS31 could regulate mitochondrial fission and fusion, inhibit mitochondrial ROS-NLRP3 activation, accelerating ATP recovery (Szeto et al., 2011; Yang et al., 2019; Yang et al., 2020). Furthermore, SS31 exerts significant podocyte protective effects in renal injury models such as aging, diabetes and high-fat mice. Nevertheless, the concrete mechanism has not been fully elucidated. In this study, we demonstrate that SS31 restores OPA1 expression by inhibiting OMA1 activation, and preserves mitochondrial function in podocyte during the progression of DKD.

## Materials and Methods

### Reagents and Antibodies

SS31 was obtained from ChinaPeptides (Shanghai, China). Streptozocin (STZ) was purchased from Sigma (Shanghai, China). Antibodies used were as follows: anti-SYNPO (NBP2-39100, Noves), anti-Nephrin (PR52265, Sigma), anti- Caspase-3 (9664s, CST), anti-OPA1 (ab42364, Abcam), anti-OMA1 (sc-515788, Santa) and anti-Tubulin (Sigma, T6074), HRP-conjugated anti-Mouse and anti-rabbit (Sigma) secondary antibody. 1,640 (11879-020) and fetal bovine serum (FBS) (A3160902) were purchased from Gibco.

### Animals

All animal care and experiments were performed according to the guidelines for the National Institutes of Health Guide for the Care and Use of Laboratory Animals and approved by the Committee on the Ethics of Animal Experiments of Nanjing Medical University, and the animal ethical approval number is IACUC-1905002 C57BL/6 male mice, weighing 18–22 g, were obtained from Charles River Laboratory Animal Technology (Beijing, China) and randomly divided into normal control group and diabetic model group. Mice from diabetic model group were intraperitoneally injected with streptozotocin (STZ) at 40 mg/kg for 3 days. Two weeks later, mice with random blood glucose higher than 16.7 mmol/L were identified as successful diabetic model mice. The experiment was divided into 2 phases. In the first phase, we observed natural pathological changes in different stages of DN. 15 diabetic mice were successfully modeled and randomly divided into 6 weeks group (n = 5), 12 weeks group (n = 5, one died accidentally halfway), and 20 weeks group (n = 5), while 4 normal mice were used as normal controls. Mice were sacrificed at corresponding time points. Blood, urine and kidney tissues were collected after euthanasia. In the second phase, we explore the podocyte protective role of SS31 in DN. 11 diabetic mice were successfully modeled and randomly divided into STZ group (n = 6) and STZ + SS31 group (n = 5), while 6 mice were used as normal controls. Mice from STZ + SS31 were intraperitoneally injected with SS31(2 mg/kg) every other day for 4 weeks, mice from control and STZ groups were correspondingly intraperitoneally injected with saline for 4 weeks. Mice were sacrificed at weeks 12. Blood, urine, and kidney tissues were collected from mice after euthanasia.

### Glomerular Harvest and Primary Podocytes Culture

Glomeruli from 8-week-old C57BL/6 mice were collected by filtering kidney tissues with different pore sizes mesh sieves. In brief, kidneys were cut into small pieces with a scalpel, immersed in 4 ml HBSS and treated with 1 mg/ml collagenase (Sigma, c6885) and 0.5 mg/ml pronase E (Sigma, p6911) at 37°C for 15 min. Then, tissues were filtrated through a 100-um cell strainer (BD Biosciences, San Jose, CA, United States) with a flattened pestle and rinsed with 15 ml HBSS. Afterward, the suspension was flown through a 400-mesh screen. Glomeruli retained on the screen were transferred with a pipette into a new 50-ml tube. Finally, the glomeruli were collected by centrifugation at 3,000 rpm for 10 min.

For primary podocyte culture, glomeruli were suspended with 1,640 containing 10% FBS and seeded on the culture dishes. The culture dishes were kept still for 3 days to allow the glomeruli to adhere. On day 4, culture medium was replaced with fresh one and the unattached glomeruli were washed away. On day 5, cellular outgrowths were detached with trypsin (Gibco) and filtrated through a 40-μm cell strainer to remove the remaining glomerular cores. The filtered cells were collected and seeded in 6-well plates at a density of 1.5×10^5^ at 37ºC with 5% CO_2_.

### Adenoviral and siRNA Transfection

6-well plates were seeded with 1.5×10^5^ cells per well. After cells adhered and reached 70–80% confluence, the corresponding volume of adenovirus was added according to the MOI and virus titer. The virus volumes were calculated by the formula “virus volume = (MOI × number of cells)/virus titer”. Cells were incubated at 37°C and the medium were replaced after 12–16 h incubation. Specific siRNA was mixed with Lipofectamine^®^ RNAiMAX (Gibco) accordingly. Briefly, cells were transfected with the siRNA- RNAiMAX complex and incubated at 37°C in a CO2 incubator for 24–96 h until gene knockdown could be detected. The mediums were changed after 4–6 h.

### Tissue Preparation and Histologic Analyses

A small piece of tissue was immersed in 10% formaldehyde solution for 1 day and then made into paraffin blocks. The blocks were cut into 3-μm sections and fixed on glass slides. Afterwards, the slides were stained with periodic acid-schiff (PAS) agent. The degree of glomerulosclerosis was semi-quantitatively calculated according to the ratio of PAS-positive areas to glomerular area using ImageJ (1.8.0).

### Immunofluorescence

A few renal cortices were frozen and cut into 3-μm thickness sections. The sections were fixed with 4% paraformaldehyde, blocked in PBS containing 10%FBS, and then incubated with primary antibodies. 40, 6-diamidino-2-phenylindole (DAPI) was used to visualize the nucleus. Finally, the samples were covered with mounting medium and observed using the fluorescence microscope.

### Transmission Electron Microscopy

Transmission electron microscopy was performed as previously described. Briefly, the kidney sections were dissected into 1 mm^3^ pieces and fixed in 3.75% glutaraldehyde. After post-fixing in 1% osmium tetroxide, samples were dehydrated in increasing concentration of alcohol, embedded in epoxy resin and then cut into 100 nm ultrathin sections. Then, sections were stained for 10 min in 2% uranyl acetate followed by lead citrate for 5 min at room temperature. Electron micrographs were obtained and analyzed using a FEI Tecnai T20 transmission electron microscope.

### Western Blot Analysis

Western blot analysis was performed as previously described (Yang et al., 2017; Wu et al., 2021). Briefly, proteins from cells and isolated glomeruli were lysed by RIPA buffer containing proteinase inhibitors and phosphatase inhibitors. After adding loading buffer, the samples were boiled at 95°C for 5 min 10 and 15% SDS-PAGE were used to separate the samples, followed by transfer of the protein from the gel to the appropriate membrane. Then, the membrane was blocked with 5% no-fat milk for 1 h, and probed with the indicated antibodies overnight at 4°C. After one night incubation, membranes were rinsed 3 times and probed with the second antibody for another 1 h. Finally, membranes were washed again and visualized using a Chemidoc Imaging System.

### Measurement of Oxygen Consumption Rate

Cells were seeded in XF24-well microplates (Seahorse Bioscience, North Billerica, MA, United States) at a density of 2.0×10^4^ cells per well. After treatment with high glucose (HG) in the presence or absence of SS31, Oxygen consumption rate (OCR) (pmol/min) was evaluated by treating cells with sequential injection of the following compounds: oligomycin (1 μmol/L), carbonyl cyanide-4 (trifluoromethoxy) phenylhydrazone (FCCP, 1 μmol/L), and antimycin A (1 μmol/L) plus rotenone (1 μmol/L). Data were normalized by protein concentration.

### Mitochondrial Morphology Staining

Cells were cultured on confocal dishes and mitochondria were labeled with the fluorescent probe Mito-Tracker Red (250 nM, Molecular Probes, Invitrogen) at 37°C for 5 min. Nucleus were labeled with DAPI. The images of mitochondrial morphology were viewed and captured using a confocal inverted laser microscope (LAM 510 Meta, Zeiss).

### Urinary Albumin Analysis

Urinary albumin was measured using a mouse albumin ELISA kit (Bethy Laboratories), Urinary creatinine was determined by the QuantiChrom Creatinine Assay kit (DICT-500, Hayward, CA) according to the manufacturer’s instructions. Data were normalized by Urinary creatinine.

### Statistical Analysis

Statistical analyses were conducted using GraphPad Prism Software. Data are presented as the mean ± SD. Differences between multiple comparisons were performed using one-way ANOVA followed by LSD test. Differences between two groups were analyzed with the Student *t* test. *p <* 0.05 was considered statistically significant.

## Results

### Podocyte Injury Is Gradually Aggravated During the Progression of Diabetic Kidney Disease

Podocyte injury plays a vital role in the development of DKD. We employed STZ to induce type 1 diabetes in mice (Supplementary Figure S1A). As shown in Supplementary Figure S1B, mice from the STZ group developed a persistent increasing microalbuminuria at weeks 12 and 20, but no significant change at week 6. PAS and WT1 staining showed that Glomeruli showed significant hypertrophy and mesangial matrix expansion at weeks 12 and 20 (Supplementary Figure S1C,D), along with abnormal nephrin distribution and decreased podocyte number (Supplementary Figure S1E,F). In addition, Electron microscopic photographs showed a marked basement membrane thickening and foot process widening at week 6 in mice from the STZ group and the slit diaphragm structures almost disappeared at week 20 (Supplementary Figure S1G–I). These results revealed that podocyte injury gradually worsened during the DKD progression.

### Mitochondrial Dysfunction in Podocyte in Diabetic Kidney Disease

**FIGURE 1:**
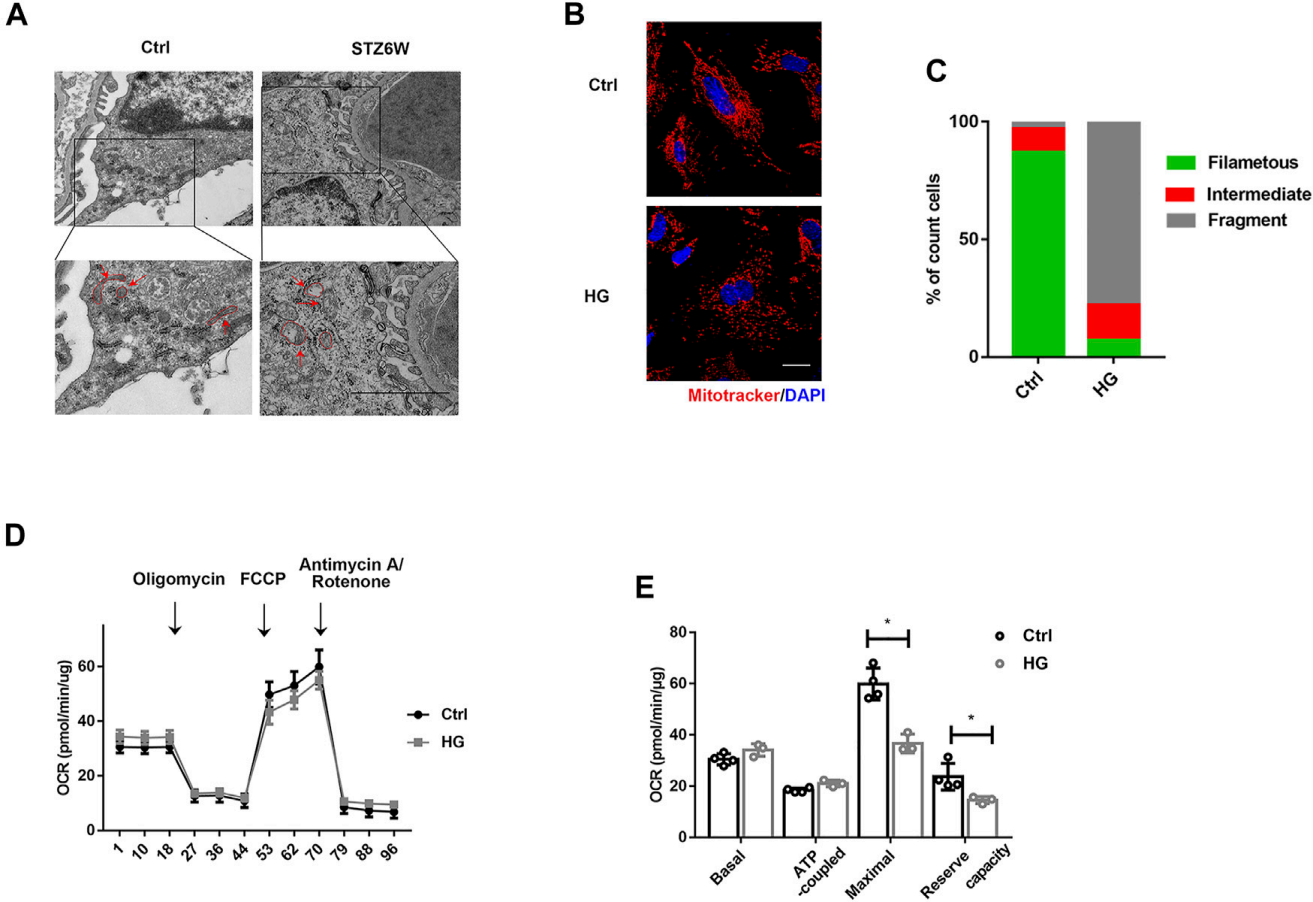
HG induces mitochondrial injury in podocytes *in vivo* and *in vitro*. **(A)** Mitochondrial morphology of podocytes in control and STZ treated mice. The red arrows indicate representative mitochondrial morphology in the groups. Electron microscopic photographs showed that mitochondria of normal podocytes were full and long, and the structure of mitochondrial cristae was clear, while mitochondrial cristae were decreased and vacuolated in podocyte from diabetic mice at weeks 6. (Bar = 2.5 μm). **(B)** Confocal images showing mitochondria. Cells in 6-well plates were challenged with HG (30 mM) for 24H and labeled with MitoTracker Red (red) and DAPI (blue). Mitochondria images were captured using a confocal microscope. As shown in the images, HG treatment shifted the mitochondria from threadiness into fragmentation. (Bar = 2.5 μm). **(C)** The histogram showing the percentage of different mitochondrial morphologies in mice with indicated genotypes. (50 fields were randomly selected for statistics). **(D)** Representative traces showing OCR in control and HG-treated podocytes. Cells in a XF24-well microplate were treated with or without HG (30 mM) for 24 h. **(E)** Statistical analyses of baseline respiratory capacity, ATP-coupled respiratory capacity, maximum respiratory capacity, and reserve respiratory capacity. n = 4,*p < 0.05: for maximal, *p* = 0.012(Ctrl vs HG); for reserve capacity, *p* = 0.03(Ctrl vs HG).

Mitochondrial dysfunction is one of the major mechanisms involved in podocyte injury and death (Hagiwara et al., 2006; Carney, 2015). Electron microscopic photographs showed that mitochondria of normal podocytes were full and long, and the structure of mitochondrial cristae was clear as indicated by the red arrow in the control group, while mitochondrial cristae were decreased and vacuolated in podocyte from diabetic mice at weeks 6 pointed out by the red arrow in the STZ group (Figure 1A). To further determine the changes of mitochondria in high glucose (HG) condition, we observed mitochondrial morphology and function in podocytes treated with HG for 24 h *in vitro*. Compared with control group, HG treatment shifted the mitochondria from threadiness into fragmentation (Figures 1B,C). Meanwhile, the mitochondrial maximal respiratory capacity and reserved respiratory capacity were both decreased under HG stimulation (Figures 1D,E). Those results further confirmed the mitochondrial dysfunction in the early stage of DKD.

### SS31 Halts the Development of Diabetic Kidney Disease

**FIGURE 2:**
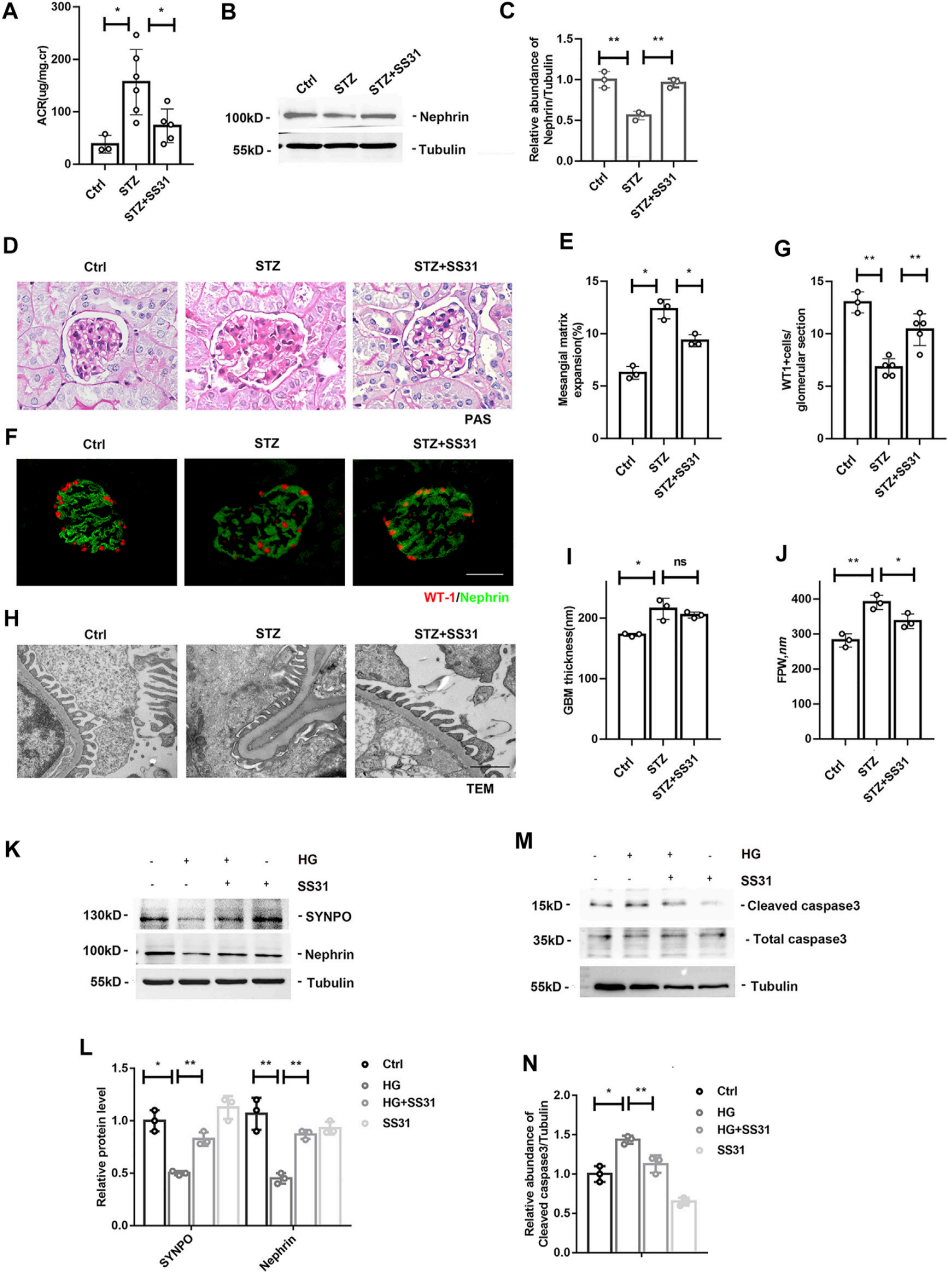
SS31 inhibits HG-induced podocyte injury. **(A)** Urinary albumin-creatine ratio of indicated groups. n = 3–6, *p < 0.05: *p* = 0.01(Ctrl vs STZ); *p* = 0.02(STZ vs STZ + SS31). **(B)** Western blot analysis showing protein expression of Nephrin in glomeruli from indicated groups at week 12 after successful induction of diabetes. **(C)** Semi-quantitative densitometry analysis for Nephrin expression. n = 3, **p < 0.01: *p* = 0.004(Ctrl vs STZ); *p* = 0.002(STZ vs STZ + SS31). **(D)** Renal histology of glomeruli by PAS staining in indicated groups. (Bar = 25 μm). **(E)** The histogram representing statistical analysis of sclerosed glomeruli in indicated groups. n = 3, *p < 0.05:*p* = 0.014(Ctrl vs STZ); *p* = 0.018(STZ vs STZ + SS31). **(F)** Representative immunofluorescent images showing WT1 and Nephrin in indicated groups. (Bar = 25 μm) **(G)** The histogram representing quantification of WT1. n = 3–5, **p < 0.01: *p* = 0.007; *p* = 0.002(STZ vs STZ + SS31). **(H)** Electron microscopic pictures of glomerular area showing podocyte foot processes and glomerular basement membrane. As shown in the TEM images, SS31 effectively improved diabetes-induced GBM thickness and foot process fusion. (Bar = 1 μm) **(I,J)** Histograms represent quantification of basement membrane thickness and foot process width. n = 3, *p < 0.05, **p < 0.01: for GBM thickness, *p* = 0.0138 (Ctrl vs STZ), *p* = 0.38(STZ vs STZ + SS31); for FPW, *p* = 0.026(Ctrl vs STZ), *p* = 0.036(STZ vs STZ + SS31). **(K)** Western blot analysis showing protein expression of SYNPO and Nephrin in podocytes cultured with high glucose in the presence or absence of SS31. Cells were pre-incubated with SS31 (100 nM) for 30 min and then HG (30 mM) for another 24 h. **(L)** Semi-quantitative densitometry analysis for SYNOP and Nephrin. n = 3, *p < 0.05, **p < 0.01: for SYNPO, *p* = 0.012(Ctrl vs HG), *p* = 0.0011(HG vs HG + SS31), *p* = 0.2(Ctrl vs SS31); for Nephrin, *p* = 0.0013(Ctrl vs HG), *p* = 0.0015(HG vs HG + SS31), *p* = 0.2(Ctrl vs SS31). **(M)** Western blot analysis showing protein expression of Cleaved caspase3 and Total caspase3 in podocyte cultured with high glucose with or without SS31. **(N)** Semi-quantitative densitometry analysis for cleaved caspase3. n = 3, *p < 0.05, **p < 0.01: *p* = 0.016(Ctrl vs HG), *p* = 0.002(HG vs HG + SS31), *p* = 0.06(Ctrl vs SS31).

We next administered SS31 to diabetic mice. After successful induction of diabetes at week 2, mice were treated with SS31. As shown in Figure 2A, SS31 significantly reduced albuminuria at week 12. Western blot analysis of glomeruli showed that SS31 rescued Nephrin downregulation in diabetic mice (Figures 2B,C). Meanwhile, PAS staining revealed that SS31 attenuated mesangial matrix expansion (Figures 2D,E). Furthermore, SS31 effectively improved diabetes-induced the loss of podocyte (Figures 2F,G) and foot process fusion (Figures 2H–J).

We further determine the effect of SS31 on HG-treated podocyte *in vitro*. Western blot analysis showed that SS31 inhibited HG-induced down-regulation of SYNPO and Nephrin (Figures 2K,L) as well as up-regulation of cleaved-caspase3 (Figures 2M,N), indicating that SS31 also ameliorates HG-induced podocyte injury *in vitro*.

### SS31 Improves Mitochondrial Function of Podocyte

**FIGURE 3:**
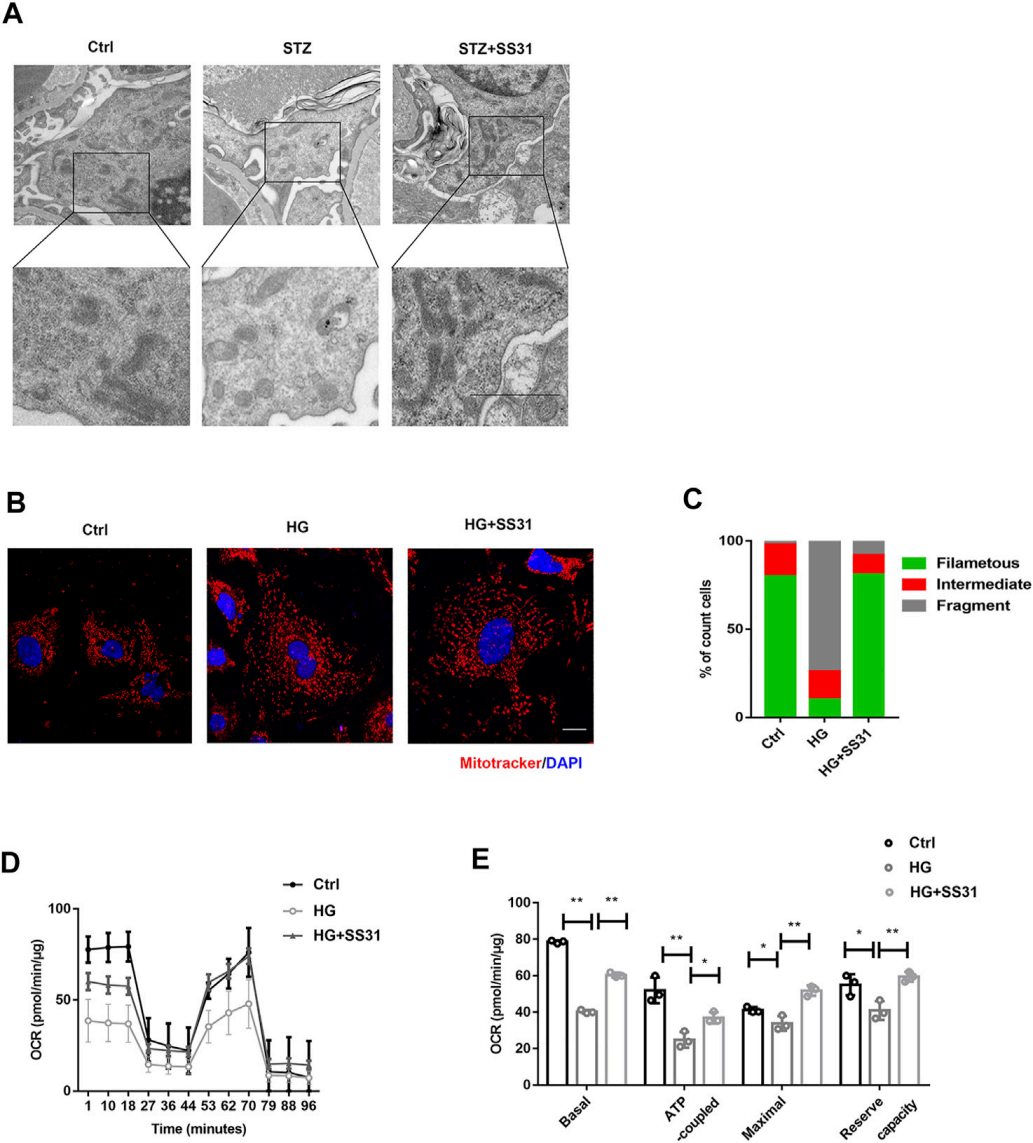
SS31 inhibits HG-induced mitochondrial injury. **(A)** Mitochondrial morphology of podocytes from control and STZ treated mice in the presence or absence of SS31. As shown in the TEM images, SS31 preserved mitochondrial cristae sharp in podocytes from diabetic mice. (Bar = 2.5 μm). **(B)** Confocal images showing mitochondria labeled with MitoTracker Red (red) and DAPI (blue). SS31 markedly ameliorated mitochondrial fragmentation. (Bar = 2.5 μm) **(C)** The histogram showing the percentage of different mitochondrial morphologies in podocytes from the indicated groups. (50 fields were randomly selected for statistics). **(D)** Representative traces showing OCR in indicated podocytes. **(E)** Statistical analyses of baseline respiratory capacity, ATP-coupled respiratory capacity, maximum respiratory capacity, and reserve respiratory capacity. n = 4. *p < 0.05, **p < 0.01: for basal, *p* < 0.001(Ctrl vs HG), *p* = 0.003(HG vs HG + SS31), for ATP coupled, *p* = 0.0048(Ctrl vs HG), *p* = 0.0172(HG vs HG + SS31), for maximal, *p* = 0.044(Ctrl vs HG), *p* = 0.0048(HG vs HG + SS31), for reserve capacity, *p* = 0.03(Ctrl vs HG), *p* = 0.003(HG vs HG + SS31).

Hence, we further examined the effect of SS31 on mitochondrial structure and function *in vivo* and *in vitro*. As shown in Figure 3A, SS31 increased mitochondria number and preserved mitochondrial cristae sharp in podocytes from diabetic mice. Additionally, SS31 markedly ameliorated mitochondrial fragmentation (Figure 3B–C) and OCR decline induced by HG *in vitro* (Figures 3D,E). All results indicated that SS31 stabilizes mitochondrial structure and function, thus protecting podocytes against HG-induced injury.

### SS31 Inhibits HG-Induced Downregulation of OPA1 in Podocytes

**FIGURE 4:**
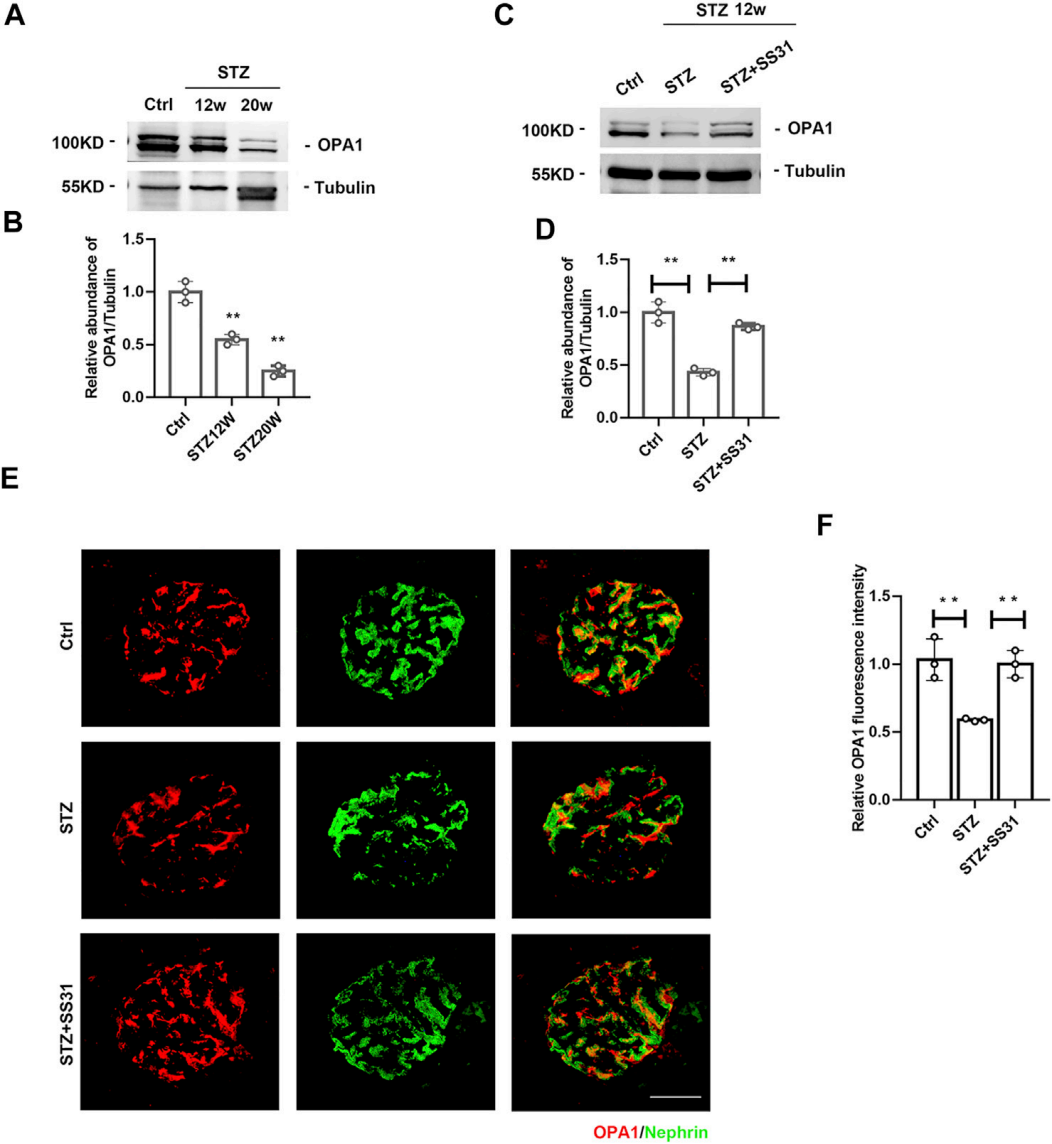
SS31 inhibits STZ-induced downregulation of OPA1 in podocytes. **(A)** Western blot analysis showing glomerular OPA1 expression in mice after STZ injection for 12 and 20 weeks **(B)** Semi-quantitative densitometry analysis for OPA1 expression. n = 3. **p < 0.01: *p* = 0.004 (Ctrl vs STZ12w), *p* = 0.002 (Ctrl vs STZ20w). **(C)** Western blot analysis showing glomerular OPA1 expression in diabetic mice with or without SS31 treatment. **(D)** Semi-quantitative densitometry analysis for OPA1 expression. n = 3. **p < 0.01: *p* = 0.002 (Ctrl vs STZ), *p* = 0.0012(STZ vs STZ + SS31). **(E)** Representative immunofluorescent images showing OPA1 and Nephrin staining in glomeruli from diabetic mice with or without SS31 treatment (Bar = 10 μm). **(F)** Semi-quantitative densitometry analysis for OPA1 fluorescence intensity. n = 3. **p < 0.01: *p* = 0.007 (Ctrl vs STZ), *p* = 0.002(STZ vs STZ + SS31).

**FIGURE 5:**
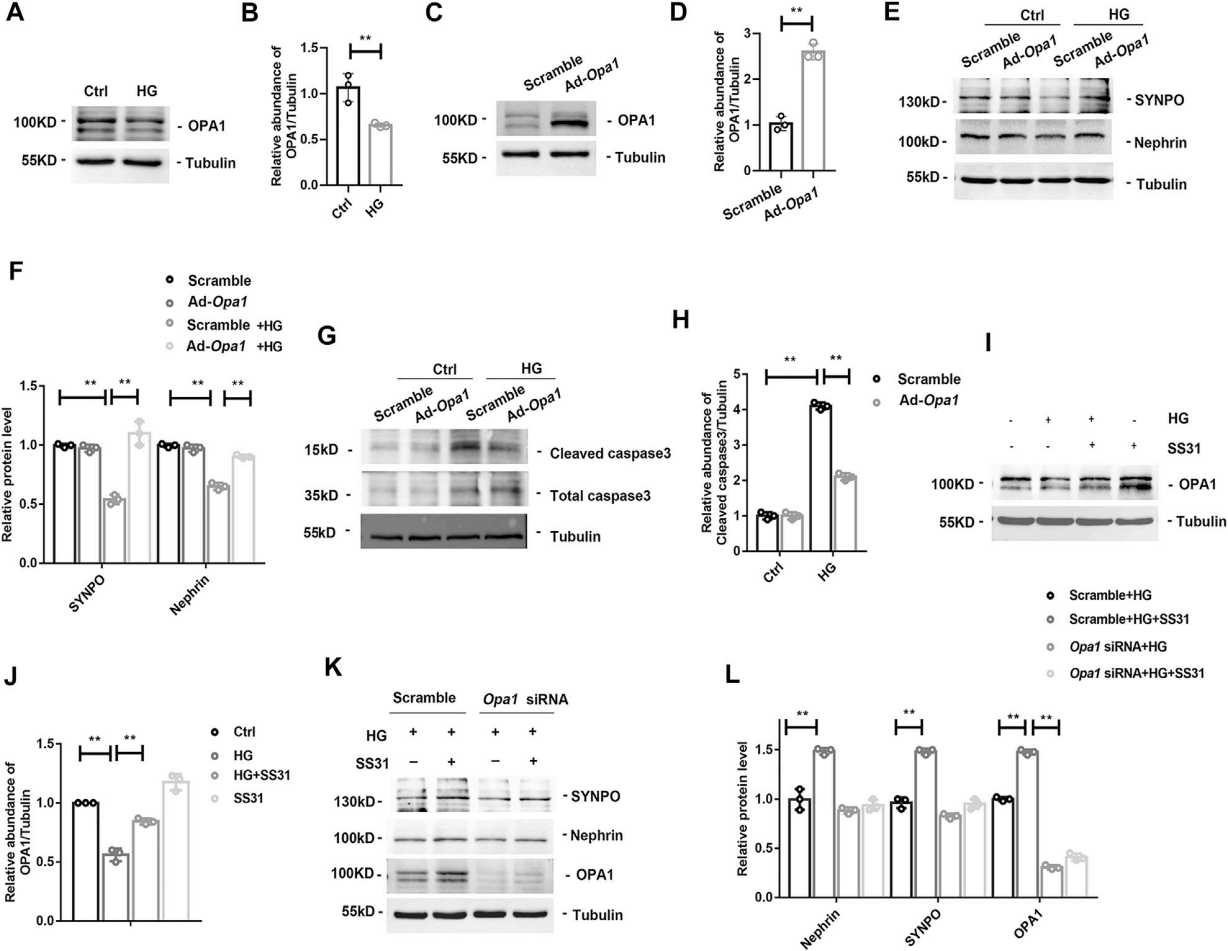
OPA1-dependent protective effect of SS31. **(A,B)** Western blot analysis showing OPA1 expression of podocytes treated with HG (30 mM) for 24H. n = 3. **p = 0.005. **(C,D)** Western blot analysis of the proteins of OPA1 in podocyte with Adenovirus-mediated *Opa1* overexpression (Ad-*Opa1*). n = 3. **p < 0.0018). **(E,F)** Western blot analysis for SYNPO and Nephrin expression in podocytes with or without overexpression of OPA1. Podocytes were transfected with adenvirus Scramble and Ad-*Opa1* for 24 h, cells were incubated with or without HG (30 mM) for another 24 h n = 3, **p < 0.01: for SYNPO: *p* = 0.004(Scramble vs Scramble + HG), *p* = 0.003(Scramble + HG vs Ad-*Opa1*+HG); for Nephrin, *p* = 0.004(Scramble vs Scramble + HG), *p* = 0.003(Scramble + HG vs Ad-*Opa1*+HG). **(G,H)** Western blot analysis showing cleaved caspase3 and total caspase3 expression in HG treated podocyte with or without Ad-*Opa1* transfection. n = 3, **p < 0.01: *p* < 0.001(Ctrl vs HG), *p* = 0.002(HG + Scramble vs HG + Ad-*Opa1*). **(I,J)** Western blot analysis showing podocyte OPA1 expression in podocytes cultured with high glucose in the presence or absence of SS31. n = 3, **p < 0.01: *p* = 0.004(Ctrl vs HG), *p* = 0.006(HG vs HG + ss31), *p* = 0.01(Ctrl vs SS31). **(K,L)** Western blot analysis for SYNPO, Nephrin and OPA1 expression in SS31 treated-podocytes cultured with HG with or without *Opa1* siRNA transfection. Podocytes were transfected with scramble and *Opa1* siRNA for 24 h. Then, cells were incubated with HG (30 mM) in the presence or absence of SS31 (100 nM) for another 24 h. n = 3. **p < 0.01: for Nephrin, *p* = 0.004(Scramble + HG vs Scramble + HG + ss31); for SYNPO, *p* = 0.002(Scramble + HG vs Scramble + HG + ss31); for OPA1, *p* = 0.002(Scramble + HG vs Scramble + HG + ss31), *p* < 0.001(Scramble + HG + ss31 vs *Opa1* siRNA + HG).

The dynamin-like GTPase OPA1 is a crucial protein that regulates the fusion of the inner mitochondrial membrane and controls mitochondrial cristae morphogenesis. The expression of OPA1 in glomeruli was gradually decreased with the development of DKD (Figures 4A,B). SS31 could halt the downregulation of OPA1 in glomeruli from diabetic mice (Figures 4C,D). Immunofluorescent staining further confirmed the effect of SS31 on OPA1 in podocyte (Figure 4E). In addition, the expression of OPA1 in podocytes was down-regulated under HG condition *in vitro* (Figures 5A,B). OPA1 overexpression significantly inhibited HG-induced SYNPO and Nephrin downregulation and podocyte apoptosis (Figures 5C–H). SS31 pre-treatment could also restore OPA1 expression in podocyte cultured with HG (Figures 5I,J). To further determine the role of OPA1 in podocyte protection of SS31 under HG condition, *Opa1* was silenced by siRNA. SS31 inhibited the HG-induced SYNPO and Nephrin downregulation, but had no effect in *Opa1* knockdown podocyte (Figures 5K,L). These data indicate that SS31 protects podocytes by stabilizing OPA1 under HG conditions.

### SS31 Inhibits the Activation of OMA1 in Podocytes in Diabetic Kidney Disease

**FIGURE 6:**
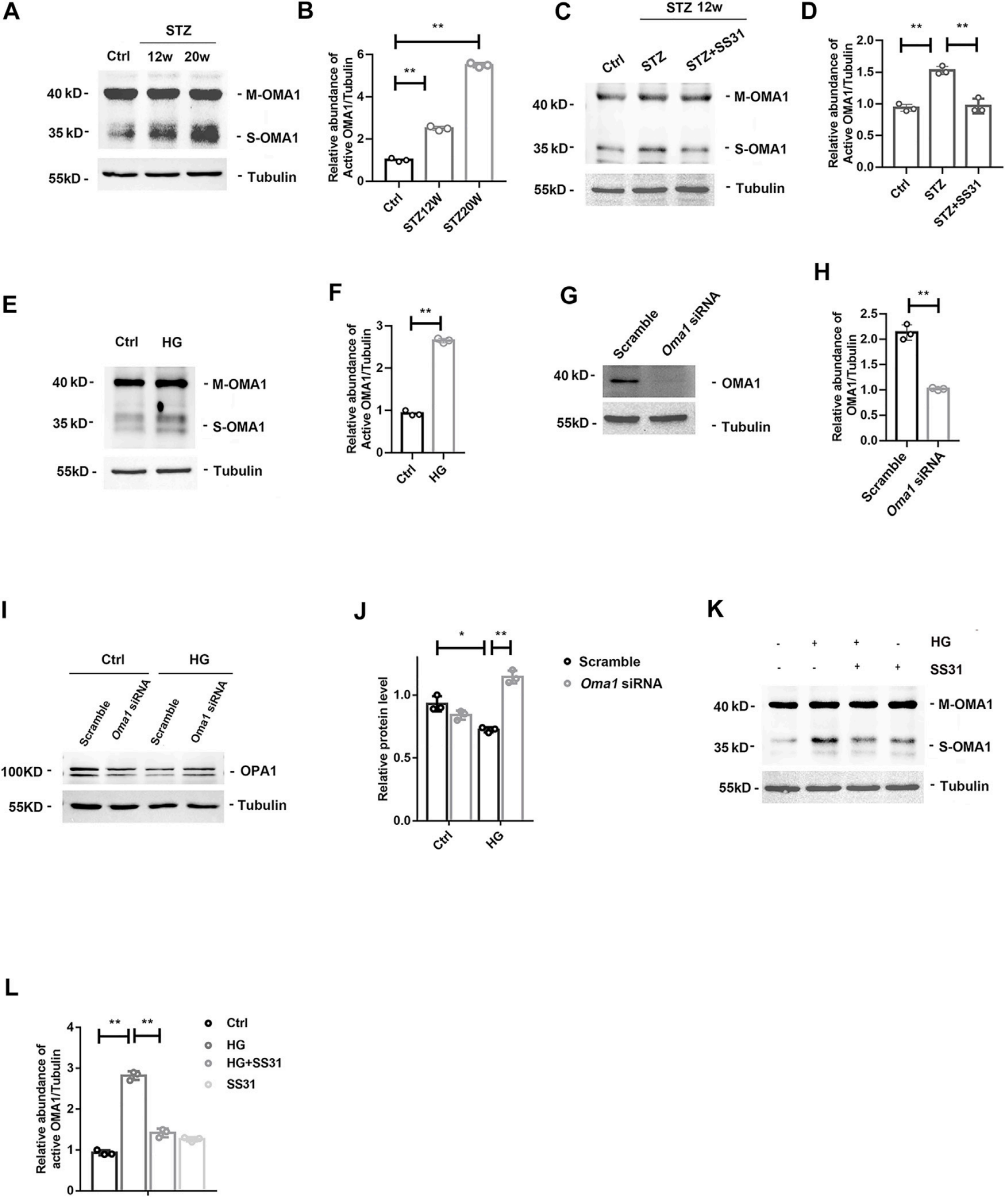
SS31 inhibits HG-induced OMA1 activation to stabilize OPA1 expression. **(A,B)**Western blot analysis showing glomerular mature form of OMA1 (M-OMA1) and short form of OMA1 (S-OMA1) expression in mice after STZ injection for 12 and 20 weeks. n = 3, **p < 0.01: *p* < 0.001(Ctrl vs STZ12w), *p* < 0.001(Ctrl vs STZ20w). **(C,D)** Western blot analysis showing glomerular OMA1 expression in diabetic mice with or without SS31 treatment. .n = 3, **p < 0.01: *p* < 0.001(Ctrl vs STZ), *p* = 0.002(STZ vs STZ + SS31)**. (E)** Western blot analysis showing mature form of OMA1 (M-OMA1) and short form of OMA1 (S-OMA1) expression of podocytes treated with HG (30 mM) for 24 h. **(F)** Semi-quantitative densitometry analysis for relative S-OMA1 expression. n = 3. **p = 0.002. **(G,H)** Immunoblot of OMA1 in podocytes transfected with or without *Oma1*-siRNA. n = 3, **p = 0.003). **(I,J)** Immunoblot of OPA1 in HG-treated podocytes transfected with or without *Oma1*-siRNA.n = 3, **p < 0.01, *p < 0.5: *p* = 0.02 (Scramble + Ctrl vs Scramble + HG), *p* = 0.006 (Scramble + HG vs *Oma1*+HG). **(K)** Western blot analysis showing M-OMA1 and S-OMA1expression of podocytes under HG in the presence or absence of SS31for 24H. **(L)** Semi-quantitative densitometry analysis for relative S-OMA1expression. n = 3. **p < 0.01: *p* < 0.001(Ctrl vs HG), *p* = 0.003(HG vs HG + ss31).

OMA1 is a mitochondrial inner membrane zinc metalloprotease and proteolytic cleave OPA1 during stress and apoptosis. We found that glomerular OMA1 was activated in a time-dependent manner in DKD (Figures 6A,B), while SS31 significantly inhibited the activation of OMA1 (Figures 6C,D). Under high glucose condition, OMA1 was activated in podocyte (Figures 6E,F). Furthermore, SS31 pre-treatment could block OMA1 activation (Figures 6G,H). We next applied *Oma1* siRNA to knockdown OMA1 expression (Figures 6I,J). *Oma1* siRNA transfected podocyte showed an increase in OPA1 expression in HG condition (Figures 6K,L). Taken together, these data indicate that SS31 can keep mitochondria fusion by inhibiting OMA1 mediated OPA1 proteolytic cleavage.

## Discussion

In this study, we reported that OMA1 activation-mediated hydrolysis of OPA1 participates in HG-induced podocyte mitochondrial injury. SS31 could inhibit the activation of OMA1to stabilize OPA1 expression, and protect podocytes from injury induced by diabetes.

Diabetes is one of the most common diseases affecting more than 350 million people worldwide, and has become an important public health challenge (Thomas et al., 2016). However, the exact molecular pathogenesis of DKD is far from being fully understood. Multiple evidence indicate that podocyte detachment is a vital factor promoting DKD development. In animal renal disease models, more than 20% loss of podocytes results in irreversible glomerular injury, manifesting as albuminuria followed by progression to ESRD (Matsusaka et al., 2005; Wharram et al., 2005). Herein, our study confirms that DKD progression is accompanied by reduced podocyte density, basement membrane thickening and foot processes flattening. As a type of terminally differentiated cells, mature podocytes have a limited capacity to proliferate in adults, susceptible to various injurious factors. Podocyte injury has become one of the major lesions leading to CKD.

Accumulated studies indicated that mitochondria play a critical role in podocyte homeostasis and progression of podocytopathy (Guan et al., 2015; Qi et al., 2017; Szeto et al., 2016; Fujii et al., 2020). Our previous study reported that compared with undifferentiated podocytes, differentiated mature podocytes have increased mitochondrial density and ATP production (Q. Yuan et al., 2020). In the present study, we discovered that mitochondrial abnormalities occur in podocytes in mice with DKD. *In vitro* studies also revealed that HG induces mitochondrial morphology abnormalities and function disorders, suggesting that podocyte mitochondrial injury may be a vital factor in the development and progression of DKD. Podocytes maintain the glomerular filtration barrier by synthesis of GBM components (Pavenstadt et al., 2003), formation of the slit membrane (Saleem et al., 2002), all of which are affected under DKD. As the results showed that diabetes leads to thickening of the GBM and swelling of the foot process. Excitingly, treatment with SS31 for 4 weeks in the initial stage of DKD exhibits significant podocyte protection and inhibits abnormalities in GBM and foot process. The promising long-term renoprotective effect of SS31 encouraged us to further explore the underlying mechanism.

Mitochondria are double-membrane organelles that form a highly dynamic network in the recurrent transformation of fusion and fission. Disruption of mitochondrial dynamics is associated with aging and various human diseases, including neurodegenerative and metabolic diseases and cancers (Bertholet et al., 2016; Chan, 2020). Mitochondrial fusion is regulated by the mitochondrial outer membrane protein MFN and the inner membrane protein OPA1 (Cipolat et al., 2004; Song et al., 2009). In addition to mitochondrial fusion, OPA1 also plays a vital role in regulating mitochondrial functions, including apoptosis and respiratory capacity (Olichon et al., 2006). Our study verified a time-dependent downregulation of OPA1 in podocyte in the progressing of diabetes. Overexpression OPA1 could ameliorate podocyte injury induced by high glucose. Those data indicate OPA1 is a crucial protein for treatment DKD.

The Szeto–Schiller (SS) peptides are cellpermeable tetrapeptides that selectively target mitochondria and concentrate on the IMM instead of penetrate into the mitochondrial matrix. Despite their 3 + net charge, they do not depolarize mitochondria as their mitochondrial uptake is potential independent (Szeto, 2017). Further study provided structural evidence for the interaction of SS31 with mitochondrial cardiolipin (CL) in liposomes, bicelles and mitoplasts. Besides inhibiting cyt c peroxidase activity, researchers have shown that SS-31 can also improve electron transfer through the cyt c/CL complex and promote mitochondrial ATP synthesis (Birk et al., 2014). Following intravenous injection of 1 mg/kg, plasma concentration of SS-31 declined rapidly with an apparent terminal half-life of about 0.8 h. SS-31 is rapidly absorbed after administration, with peak plasma levels detected within 15 min. Bioavailability of SS-31 after subcutaneous administration was higher in the dog (72.7%) and monkey (81.4%) compared to rat (38%) (Szeto and Schiller, 2011). SS31 has a wide range of renal protective effects, including podocytes. It has been reported that SS31 is capable of restoring C3a-induced podocyte motility (Morigi et al., 2020), preserves podocyte number and foot process in mice fed a high-fat diet (Szeto et al., 2016), and inhibits mitochondrial oxidative injury and Cyt c release and elimination of mtROS, thus preventing the activation of podocyte apoptosis pathways in diabetic rats (Wang et al., 2019)and improving podocyte cytoskeletal integrity in mice of advanced age (Sweetwyne et al., 2017). Consisting with previous studies, SS31 has benefit on podocyte in mice with diabetes. The mitochondrial function also is improved by SS31 treatment by restore OPA1 expression.

OMA1 is a distinct metal endopeptidase with little protease activity under physiological conditions but activated upon mitochondrial stress. In response to stress, the C-terminal self-cleavage of OMA1 can promote its activity (Zhang et al., 2014). The substrates of OMA1 include DELE1 and OPA1 (Alavi, 2021). OMA1 controls the dynamic balance of mitochondrial fission and fusion by regulating the hydrolysis of OPA1 (MacVicar and Langer, 2016). OMA1 activity is enhanced by a variety of stresses including accumulation of unfolded polypeptides and dissipation of the membrane potential as well as ROS (Richter et al., 2015). In this study, HG can activate OMA1 which further hydrolyzes OPA1, meanwhile SS31 can protecting podocytes by inhibiting the activation of OMA1 to stabilize OPA1 expression. In the study, we did not exclude the effect of osmotic pressure *in vitro*. However, as shown in Supplementary Figure S2, mannitol had no significant effect on Nephrin, OPA1 and OMA1 protein expression in cultured podocytes, while high glucose significantly changed the expression of these molecules, suggesting that the changes *in vitro* were specifically caused by high glucose concentration rather than high osmolality.

In summary, OPA1 plays an important role in podocyte mitochondrial morphology and function, we confirmed that SS31 protects podocytes in DKD via inhibiting OMA1-mediated hydrolysis of OPA1. Our study provides a new insight into the mechanism of SS31 in protecting podocyte mitochondria under diabetes, indicating that SS31 might be a promising agent in delaying the progression of DKD.

### Study Limitation

To better mimic the situation of cells *in vivo*, primary podocytes were performed in our *in vitro* experiments. We utilized filters with different pore sizes to isolate the glomeruli from kidney and acquire primary podocytes. Theoretically, we can completely separate other cells such as macrophages from glomeruli using filters with different pore sizes according to different cell diameters. However, a small number of cells may inevitably aggregate and remain in our extracted cells, resulting in the purity of podocytes that cannot reach 100%, and we could not exclude the effect of the remaining small fraction of cells on the experiment. We tested the purity of our cells above 90% by flow cytometry. Although magnetic beads or flow sorting can further improve the purity of cells, they are costly and the current technology cannot still reach 100% purity. We chosed the affordable approach. The effect of the minimal other cells on experiments was hard to exclude. Maybe more advanced and affordable techniques are needed to improve the purity of primary cells in the future.

## Data Availability

The raw data supporting the conclusions of this article will be made available by the authors, without undue reservation.
