# Supplementary figures and images for: SS31 Ameliorates Podocyte Injury via Inhibiting OMA1-Mediated Hydrolysis of OPA1 in Diabetic Kidney Disease
Source: Front Pharmacol. 2022 Jan 31;12:707006. doi: 10.3389/fphar.2021.707006 (PMC9629008; doi:10.3389/fphar.2021.707006)

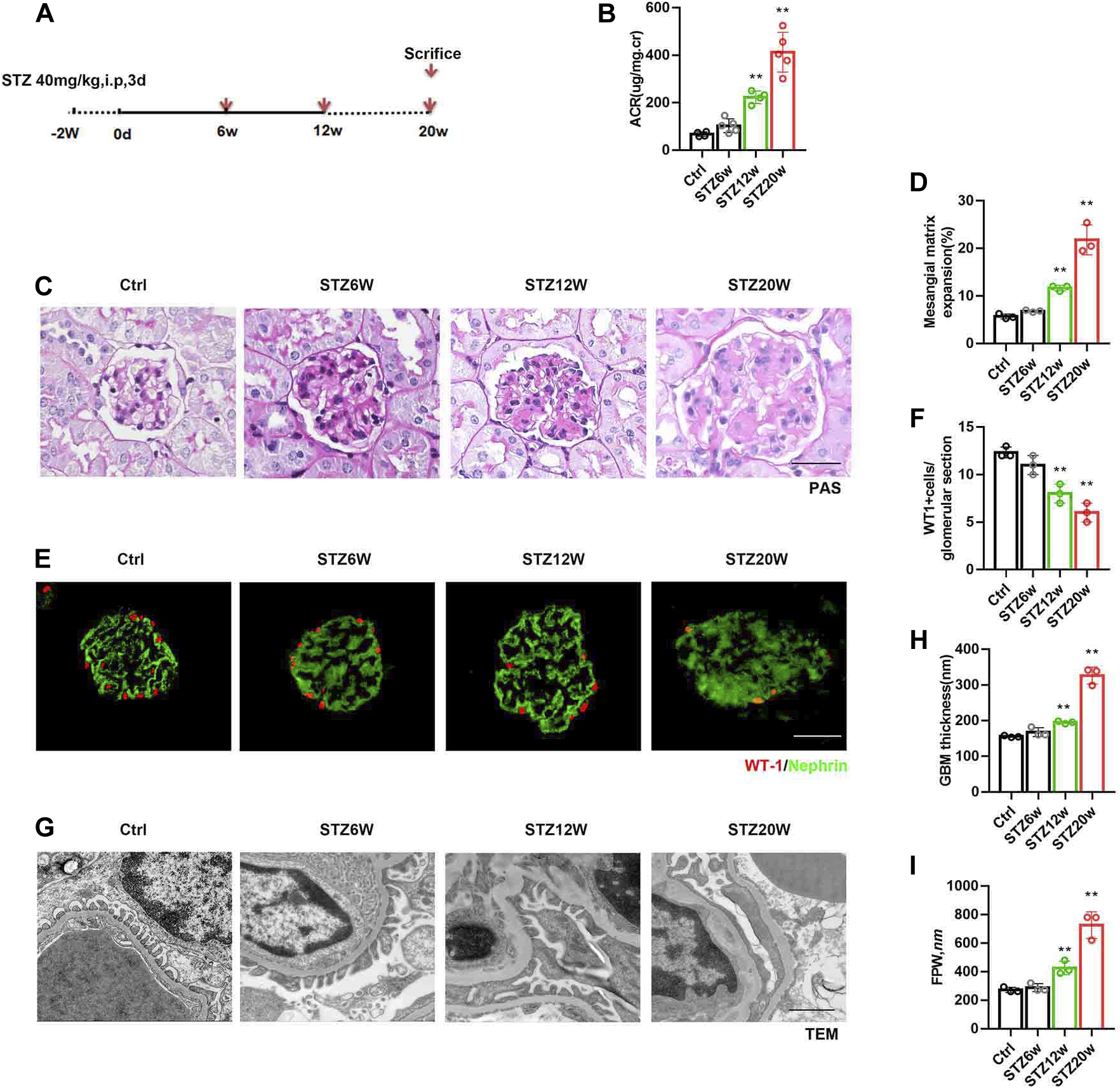

Supplement: Supplementary file 1 [file Image1.tif]

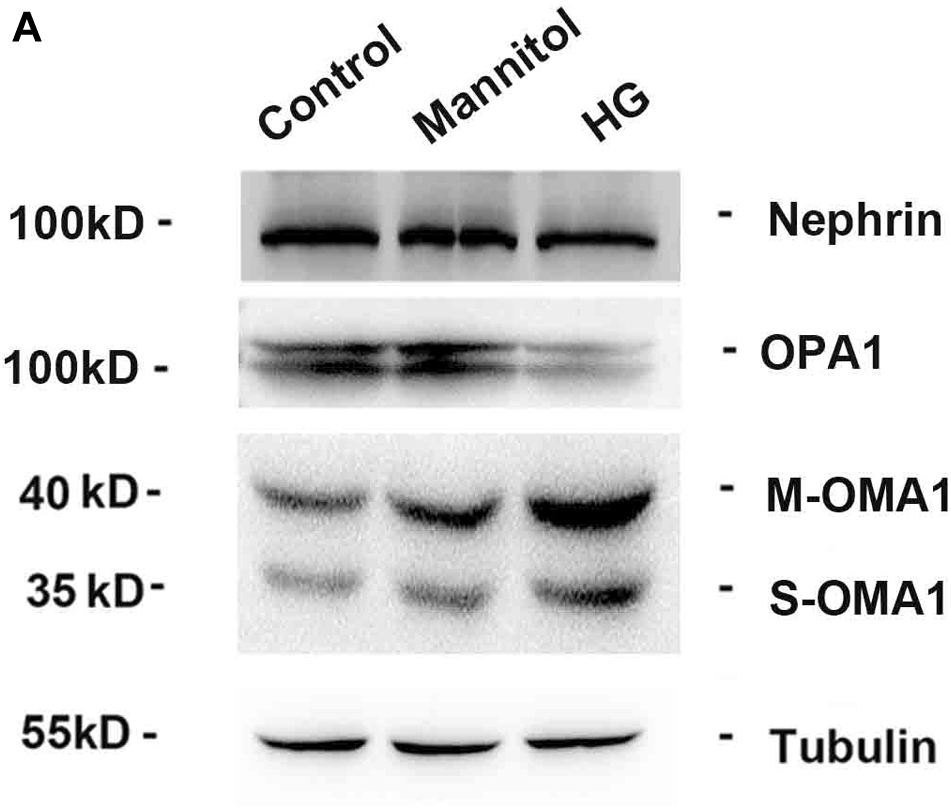

Supplement: Supplementary file 2 [file Image2.tif]
